# Molecular Marvels: Small Molecules Paving the Way for Enhanced Gene Therapy

**DOI:** 10.3390/ph17010041

**Published:** 2023-12-27

**Authors:** Sebastian Hasselbeck, Xinlai Cheng

**Affiliations:** 1 Buchmann Institute for Molecular Life Sciences, Goethe University Frankfurt am Main, 60438 Frankfurt am Main, Germany; hasselbeck@pharmchem.uni-frankfurt.de; 2 Institute of Pharmaceutical Chemistry, Goethe University Frankfurt am Main, 60438 Frankfurt am Main, Germany; 3 Frankfurt Cancer Institute, 60596 Frankfurt am Main, Germany

**Keywords:** Cas9, small molecules, genome editing

## Abstract

In the rapidly evolving landscape of genetic engineering, the advent of CRISPR-Cas technologies has catalyzed a paradigm shift, empowering scientists to manipulate the genetic code with unprecedented accuracy and efficiency. Despite the remarkable capabilities inherent to CRISPR-Cas systems, recent advancements have witnessed the integration of small molecules to augment their functionality, introducing new dimensions to the precision and versatility of gene editing applications. This review delves into the synergy between CRISPR-Cas technologies based specifically on Cas9 and small-molecule drugs, elucidating the pivotal role of chemicals in optimizing target specificity and editing efficiency. By examining a diverse array of applications, ranging from therapeutic interventions to agricultural advancements, we explore how the judicious use of chemicals enhances the precision of CRISPR-Cas9-mediated genetic modifications. In this review, we emphasize the significance of small-molecule drugs in fine-tuning the CRISPR-Cas9 machinery, which allows researchers to exert meticulous control over the editing process. We delve into the mechanisms through which these chemicals bolster target specificity, mitigate off-target effects, and contribute to the overall refinement of gene editing outcomes. Additionally, we discuss the potential of chemical integration in expanding the scope of CRISPR-Cas9 technologies, enabling tailored solutions for diverse genetic manipulation challenges. As CRISPR-Cas9 technologies continue to evolve, the integration of small-molecule drugs emerges as a crucial avenue for advancing the precision and applicability of gene editing techniques. This review not only synthesizes current knowledge but also highlights future prospects, paving the way for a deeper understanding of the synergistic interplay between CRISPR-Cas9 systems and chemical modulators in the pursuit of more controlled and efficient genetic modifications.

## 1. Introduction

In bacteria and archaea, an important part of their immune systems are the clustered regulatory interspaced short palindromic repeats (CRISPRs) [1]. These are utilized to protect the host organism from invading viruses and plasmids. Within this system, the nucleic acids of intruders are silenced by specific small ribonucleic acids (RNAs) originating from the host organism itself [2]. Over recent years, scientific advancements have transformed this system into a practical tool for (epi)genome editing, organismal studies, and the exploration and combat of diseases, particularly hereditary diseases [3]. Playing a pivotal role in the CRISPR system are the CRISPR-associated proteins (Cas) [4]. Among them, Cas9 is the most widely used [5]. It functions as an endonuclease, capable of recognizing specific double-stranded DNA (dsDNA) and, in turn, silencing it through cleavage [2]. Moreover, in scientific applications, after dsDNA cleavage, a new sequence can be inserted to achieve designated genome editing [3]. However, low editing efficiency and unwanted off-target effects largely limit clinical applications of the CRISPR/Cas9 system [6]. This overview aims to delineate the potential influence of small molecules on the CRISPR/Cas9 system. While there are comprehensive reviews about modulating Cas9’s activity via small molecules, they mainly focus on indirect mechanisms. Chen et al. discussed in a detailed manner the impact of small molecules on the HDR pathway, DNA ligase IV inhibitors, and anti-CRISPR small molecules, -peptides, and -nucleic acids. The discussion of direct interactions between small molecules and Cas9, however, felt short [7]. A further review conducted by Shams et al. discusses small molecule-based influence on Cas9 in a very similar way, focusing on DNA repair mechanisms and influencing the multiple important factors during this process [8]. While both contribute an important part to a better understanding of said topics and a great overview of scientific advancements in those fields, those topics will be discussed rather briefly in this review. A bigger focus will be directed towards immediate interactions between small molecules and Cas9, be it wild-type or modified.

## 2. Genetic Structure and Function of the CRISPR System

**Figure 1:**
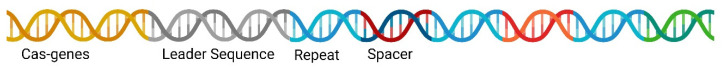
The general structure of a CRISPR locus. Consisting of multiple Cas genes organized in operons (shown in yellow), followed by a leader sequence (gray), and afterwards the repeat (blue) array in which the spacers (various colors) are built in. The various colors indicate that the spacers are of different nature, while the consistent blue color indicates the consistent nature of the repeats. Furthermore, the consistency of length is shown [2,9].

The CRISPR gene locus consists of Cas genes, a leader sequence, short repeats, and similar short spacers positioned between the repeats. The length of the repeats varies from 21 to 47 bp, contingent on the species, while remaining constant within a species. The genetic structure of a CRISPR gene locus is shown in Figure 1 [9].

**Figure 2:**
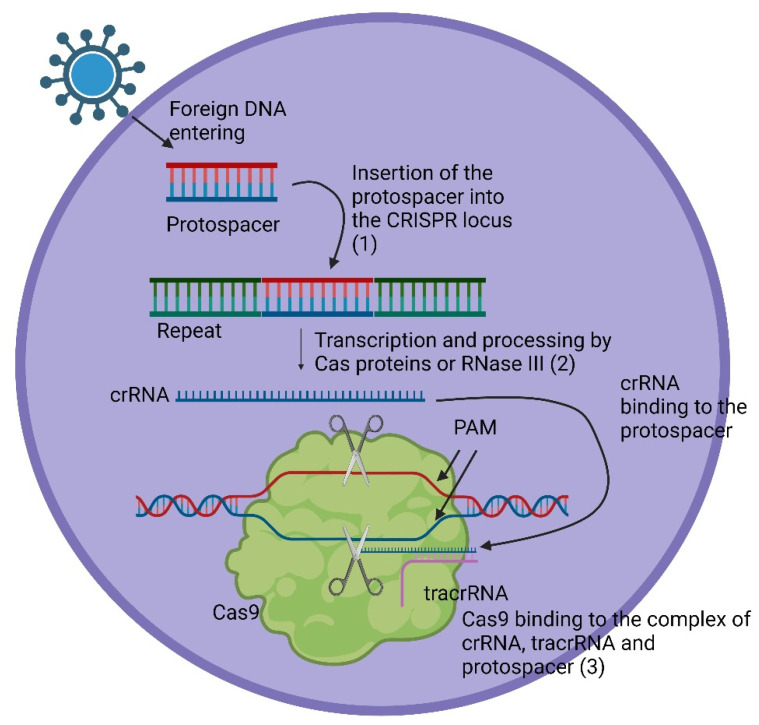
The general triad of an immune reaction within the CRISPR system. In the adaptive phase, (1) foreign DNA enters the cell, and the protospacer is integrated between two repeats. In the expression phase (2), crRNA is transcribed and further processed by Cas proteins (Systems I and III) and then binds onto the protospacer of the foreign DNA. In case of System II, this happens with the help of a tracrRNA. The formed complex, in the case of system II, consisting of Cas9, crRNA bound to the protospacer of the viral DNA, and tracrRNA bound to the repeat sequence of the crRNA, leads to silencing of the foreign DNA through a DSB at the PAM by the endonuclease activity of Cas9 [1,2].

Importantly, the spacers do not originate from the host organism but from intruding viruses or plasmids. These spacers, integral parts of the genetic material of the intruders, are incorporated as proto-spacers between the repeats during the initial adaptive phase (1) of the immune reaction. This integration occurs when a bacterium or archaea encounters an intruder for the first time. The spacer is always introduced at the proximal end of the CRISPR array [2]. Subsequent to this adaptive phase, two additional phases unfold in the immune response. In the expression phase (2), CRISPR RNAs (crRNAs) are expressed, containing both the repeat sequence and the spacer sequence. For instance, a Cpf1 (also known as Cas12a) crRNA contains 42–44 nt, with a 19 nt-repeat and a 23–25 nt-spacer [10]. In the interference phase (3), crRNA pairs with the complementary proto-spacers of an invading virus or plasmid, facilitating the recognition and subsequent silencing of the intruding genetic material by Cas proteins [2]. The position of the double-strand break (DSB) is determined by the complementary pairing and the location of the proto-spacer adjacent motif (PAM) [2], with the PAM sequence being 2–5 nt long and specific to certain bacteria [11]. Similar to Cas9, other Cas proteins can act as endonucleases, cutting the DNA strands near the binding position of the proto-spacer [12]. There are three types of systems in which DNA cutting can occur. In systems I and III, pre-crRNA is processed by Cas proteins, and the complementary strand is then detected and cleaved in a multi-enzyme complex. In system II, pre-crRNA initially binds to trans-activating crRNA (tracrRNA), which is complementary to the repeats, and processing is conducted by RNase III in the presence of Cas9 [2]. The process of an immune reaction is shown in Figure 2. 

**Figure 3:**
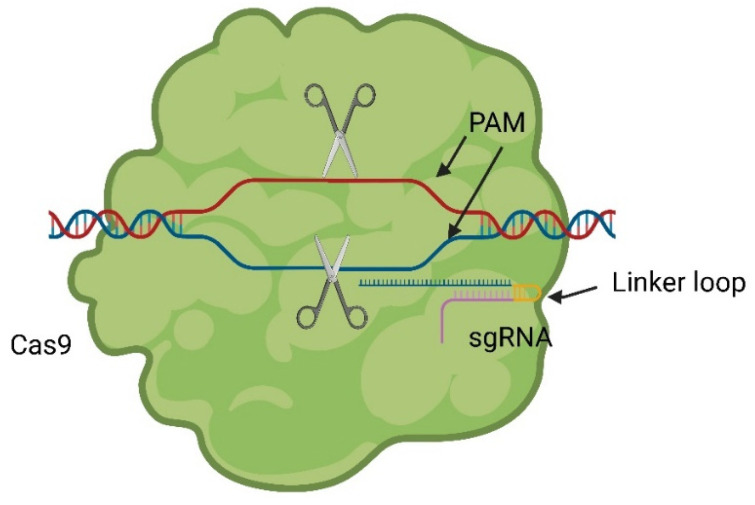
The sgRNA combines tracrRNA and crRNA into one ribonucleic acid and induces the same effect as the complex shown in Figure 2 [2].

By producing a chimera of tracrRNA and crRNA, connected through a linker loop at the 3′ end of the crRNA, a single guiding RNA (sgRNA) can be formed. This allows Cas9 to be precisely programmed for inducing a DSB at a specific genomic position using single RNA [2]. The underlying principle closely mimics the naturally occurring process, as illustrated in Figure 3.

## 3. CRISPR/Cas9-Mediated Genome Editing

**Figure 4:**
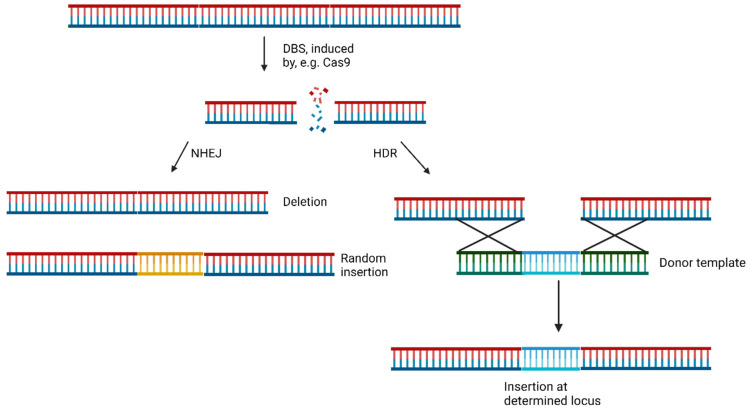
The pathways of NHEJ (left) and HDR (right). During an NHEJ after a DSB, a part of the genetic material is lost, or a random genetic modification (orange) is included. During an HDR, a donor template (green and blue) hybridizes with the broken DS, and new genetic information (blue) can be precisely inserted [3,14,15,16].

The widely studied CRISPR/Cas system, derived from *Streptococcus pyogenes* (spCas9), is predominantly employed for genetic editing. This Cas9-based system falls under type II of the CRISPR systems [13]. During the genome editing process, a single-guide RNA (sgRNA) directs Cas9 to a specific location to induce a DSB. Non-homologous end joining (NHEJ), and homology-directed repair (HDR) are two major pathways for DSB repair. The more prevalent and efficient NHEJ pathway does not require a template [3]. It leads to either the deletion of genetic information (knockouts) or the introduction of random genetic modifications (collectively termed indels) into the DSB [3,14,15]. In contrast, HDR can facilitate the precise incorporation of genetic information by pairing the broken strands with a new complementary strand at the ends. This allows for the hybridization of strands and the insertion of the strand containing the new genetic information [16]. NHEJ is present in all four cell cycle phases, while HDR is mainly limited to phases S and G2, when DNA duplication has occurred, and sister chromatids are available for repair [17]. The mechanics of NHEJ and HDR are shown in Figure 4 [3]. Moreover, microhomology-mediated end joining (MMEJ), a subtype of NHEJ, involves annealing of two 3′-ends of a DSB at microhomologies, leading to 3′ flaps that are removed, and the strands are subsequently filled in. Notably, this process leads to significant loss of genetic information due to flap removal, resulting in deletions known as microhomologies containing deletions (MH) [18].

To introduce a new gene, a substantial amount of the donor template is required, primarily favoring the homology-directed repair (HDR) pathway when the correct template is available [11]. This process, known as knock-in (event), involves the precise incorporation of a new gene [17]. To deliver the CRISPR/Cas-9 system into cells, various methods have been developed, including physical approaches, such as injection, or viral delivery methods using, for example, adenoviruses or non-viral delivery methods like liposomes [19]. 

**Figure 5:**
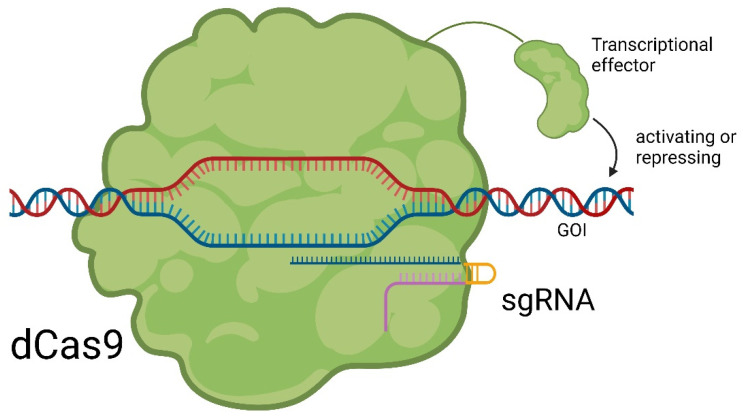
Principle of epigenetic editing with dCas9. Catalytic unfunctional dCas9 binds to the promotor region of a GOI. The effector attached to dCas9 either activates or represses the gene, leading to an altered expression [25].

Besides the genomic editing by inserting full donor templates that can be expressed, it is possible to make an endonuclease-dead Cas9 (dCas9) by two-point mutations, H840A and D10A, which lead to the loss of the endonuclease activity [20]. In this configuration, dCas9 can still bind to a specific DNA sequence under the guidance of designated sgRNA. However, due to the absence of endonuclease activity, target dsDNA is kept intact. When modified with gene effectors and targeting the promoter/enhancer region of the gene of interest (GOI), dCas9 can either activate or repress the GOI transcription, termed CRISPRi (CRISPR interference) and CRISPRa (CRISPR activation) [21,22]. Examples of gene effectors include Kruppel-associated box (KRAB) for repression [23] and Herpes simplex viral protein (HSVP) for activation [24]. The principle of epigenome editing with dCas9 is shown in Figure 5 [25]. 

Further applications include single nucleotide exchanges or the introduction of new protospacer adjacent motifs, enabling broader utilization within the human genome [3]. 

The applications of genetic editing with CRISPR/Cas9 span from human disease model and diagnosis to gene therapy [3]. Mainly inherited and genetically based diseases are tried to be treated, such as blood, eye, muscular, and further disorders [26]. As an example, for a genetic disease, there is β-thalassemia. In this case, scientists try to reactivate fetal γ-globin via the correction of mutations [27]. For example, Métais et al. were able to increase γ-globin by silencing the *BCL11A* gene, which itself is a transcriptional repressor of γ-globin [28]. Besides genetic applications for humans, plants can be addressed as well. There are many traits of crops, for example, that can be influenced to yield a better product. Those can be of external (shell, size, color) or internal (nutritional values, e.g., a high amount of protein) nature [29]. By modifying the quantitative trait loci (QTL), Shen et al. were able to increase the size of several rice crops in comparison to the wild types of multiple *japonica* variants [30].

Given the diverse methods in the realm of genes, it is crucial to control Cas9’s action in terms of dosage, temporal aspects, and spatial considerations [31,32]. This control is essential for achieving reproducible results and, more importantly, ensuring the safety of Cas9 gene editing in therapeutic applications [6]. The significance lies in the fact that Cas9 has been shown to exhibit several side effects with prolonged activity at high levels, including off-target genome editing, gene toxicity, gene translocation, and more [31]. One approach to inhibiting Cas9 involves naturally occurring proteins from bacteriophages, developed as a counter to CRISPR systems [33]. Some of these, such as AcrIE1, have demonstrated the ability to block site-specific DNA cleavage [34]. However, therapeutic proteins come with significant drawbacks, including a short half-life, poor stability, low solubility, and high production costs [35]. This underscores the interest in identifying small molecules that can be employed to control Cas9 activity [6]. Small molecules are often more cost-effective, better soluble, more stable, easier to produce and modify, and cheaper to manufacture [36].

## 4. Small Molecules Modulate Wild-Type Cas9 Protein

Through high-throughput screening, Maji et al., identified several small molecules that could disrupt Cas9 binding to DNA, preventing DNA double-strand breaks (DSBs). Testing eGFP further confirmed that some of these Cas9 inhibitors, such as BRD0539 (**1**), worked reversibly [37,38]. Recently, using a high-content fluorescence-based approach, we identified valproic acid (**2**) (VPA) as a Cas9 degrader from a chemical library consisting of nearly 300 drug-like compounds and natural products [39]. VPA, a well-known histone deacetylase inhibitor (HDACi), demonstrated significant Cas9^WT^ degradation under hyperthermia conditions generated either in the presence of a photothermal agent, indocyanine green, upon irradiation by a near-infrared laser or heating with an external heat bag. This degradation effect was independent of its HDAC inhibitory effect. However, the off-target effects of BRD0539 or VPA remain unknown.

**Figure 6:**
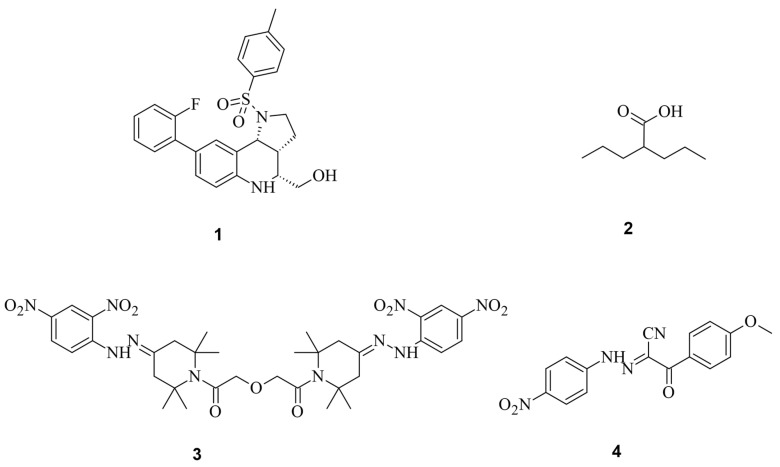
The spCas9 small molecules inhibitors and degraders BRD0539 (**1**), VPA (**2**), SP24 (**3**), and SP2 (**4**) [37,38].

For a clearer understanding of off-target effects, Yang et al. searched for small molecules that inhibit Cas9^WT^. In their studies, the most effective Cas9 inhibitor was shown to be SP24 (**3**), with an IC_50_ for Cas9^WT^ of approximately 14 µM and for the Cas9-sgRNA complex of about 7 µM. This was shown during an FP assay, where SP24 significantly decreased fluorescence polarization [38]. While there are very much more potent inhibitors (nM range) [40], the SP inhibitors showed higher IC_50_ values for Cas9^wt^ than the previously known BRD0539 inhibitor with an IC_50_ of 22 µM [37]. And even though the IC_50_ may not be optimal, the advantage is that the inhibitor can be applied to Cas9^wt^. The upcoming discussions will emphasize the often-severe modifications of Cas9 to make it addressable by small molecules. Furthermore, SP24 and SP2 (**4**) were proven to enhance the precision of Cas9-mediated genome editing (Figure 6) [37,38].

## 5. Small Molecules Modulate Engineered Cas9 Protein 

**Figure 7:**
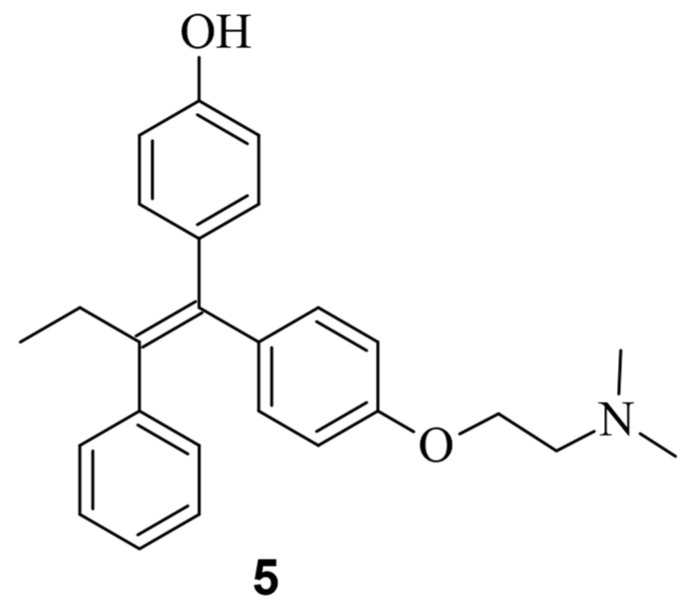
Chemical structure of the small molecule 4-hydroxytamofen (**5**).

While only a few small molecules have been identified to function with Cas9^WT^, the primary Cas9 chemical modulators interact with engineered Cas9. A way to activate Cas9 with a small molecule was demonstrated by Davis et al. [41]. As shown in Figure 7, Buskirk et al. successfully evolved inteins that could only self-remove in the presence of 4-hydroxytamoxifen (4-HT) (**5**) [42]. 

**Figure 8:**
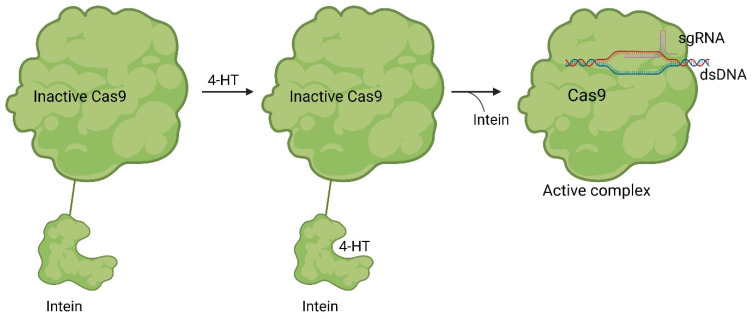
Working mode of intein inhibited and activated Cas9. Through a modification with an intein, Cas9 is inactivated (left). Through treatment with the small molecule 4-HT, self-splicing of the intein is induced (middle). After splicing, Cas9 can form an active sgRNA complex, and the desired gene can be modified [41].

Upon binding to the intein, 4-HT induces the self-splicing of the intein [42]. In the case of Cas9, it is inactivated when the intein is attached to it. However, treatment with 4-HT led to the self-splicing of the intein from Cas9, reactivating its functionality (Figure 8) [41].

Davis et al. modified spCas9 at 15 different positions and expressed the modified Cas9 variants in HEK293-GFP cells with sgRNA targeting the *EGFP* locus. They determined the loss of function in expressing GFP after treatment with 1 µM 4-hydroxytamoxifen in 8 cases. In a more in-depth analysis with Cas9 variants modified at S219 and C574, respectively, in comparison to Cas9^wt^, it was demonstrated that the modified variants exhibited higher specificity at similar on-target cleavage rates. For instance, the C574-modified Cas9 and Cas9^wt^ had similar on-target DNA cleavage rates of 6.4%. However, the modified variant resulted in a fourfold lower frequency at the four critical off-target sites [41]. By precisely activating Cas9, the precision of Cas9-mediated genome editing could be significantly enhanced. 

**Figure 9:**
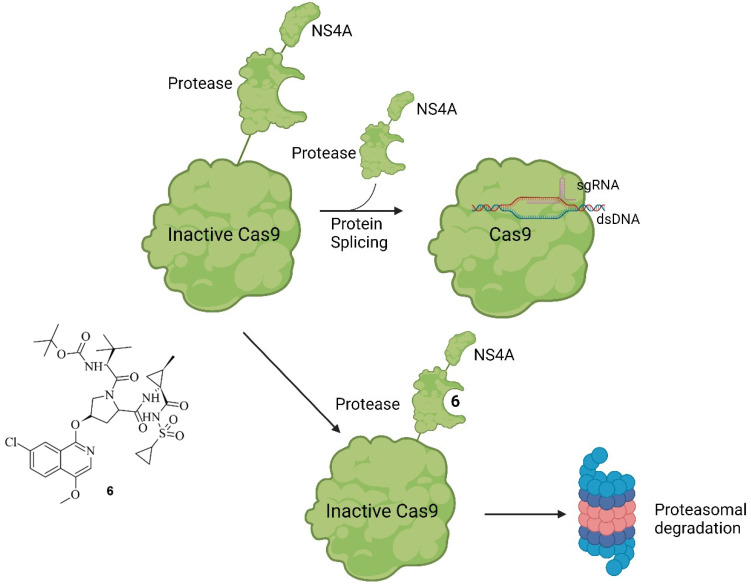
Working principle of SMASh tag-controlled Cas9. Under non-treatment conditions, modified Cas9 will be processed into active Cas9 by self-cleavage of the SMASh tag. The tag will be degraded by the proteasome or lysosome. If, however, treatment with ASV (**6**) (shown on the left) is applied, the protease activity is inhibited, and Cas9 marked with a SMASh tag is degraded as a whole unit [43].

A similar approach was undertaken by Wu et al., where Cas9 was modified with a small molecule-assisted shut-off (SMASh) tag. Cas9 was fused with a degron from the hepatitis C virus (NS4A) and a protease domain. Under non-treatment conditions, the protease self-cleaves the SMASh tag, converting Cas9 into an active species. However, upon adding the protease inhibitor Asunaprevir (ASV) (**6**), the protease activity is inhibited, preventing the cleavage of the SMASh tag. This results in the recognition of the degron by the proteasome or lysosome, leading to the degradation of Cas9 and rendering it inactive. With this system, the gene editing specificity in comparison to Cas9^wt^ could be increased by 1.4-fold in the lowest case (*EMX1*) up to 8.7-fold in the highest case (*VEGFA*) while targeting different genes with an application of 20 µM of ASV. The mode of action of this system, as well as ASV, are shown in Figure 9 [43]. 

**Figure 10:**
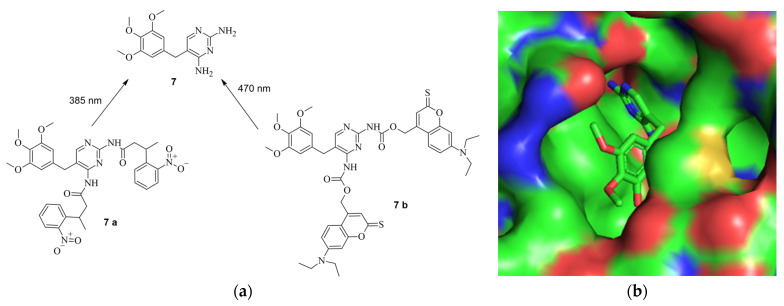

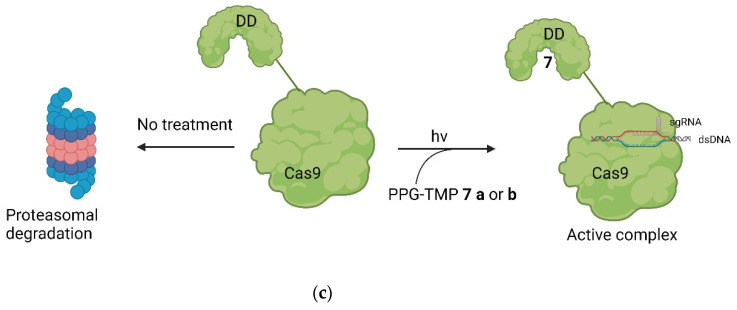
(**a**) (above, left): The structure of TMP (**7**) and the protected derivates **7 a** and **7 b**, including the used wave lengths for deprotection [32]. (**b**) (above, right): The structure of TMP in the binding pocket of DHFR. The amines are deep in the binding pocket, leaving them a good target for protection (PDB: 7R6G) [32,48]. (**c**) (below): The principle of the system. Through introduction of the DDs, Cas9 is quickly degraded by the proteosome. By treatment with PPG-TMP and irradiation (hv), free TMP can be formed, bind to the DDs, stabilize them, and thus turn Cas9 active [32].

The working principle is exactly inverted in comparison to the system described earlier. In the first case, the addition of a small molecule activates Cas9 through cleavage, as demonstrated by Davis et al. [41]. In the second case, the addition of the small-molecule inhibitor prevents self-cleavage and keeps Cas9 inevitably inactive because it is degraded by the proteasome or lysosome [43]. Notably, the removal of ASV by washing with uncontaminated media allowed the newly expressed Cas9 to become active again since self-cleavage was not hindered anymore. This reversibility is valuable to prevent Cas9 from re-editing previously edited loci [44]. Often, the editing of multiple genes is necessary [45], and in such cases, Cas9 can be inactivated after editing a particular gene and re-activated when editing the next gene. Such a light-switch-like system is extremely useful for controlling the effects of Cas9, making it safer and more efficient [3,43]. Speaking of light, it represents a very useful tool for spatial control of the activity of small molecules [46]. Based on photoactivable protecting groups (PPGs), Manna et al. designed a “fused” system of the ones described by Davis et al. and Wu et al. [41,43]. Cas9 was modified with destabilized domains (DDs) of dihydrofolate reductase (DHFR). Through these unstable domains, the fused Cas9 is recognized and degraded rapidly by the proteasome, making it inactive, similar to the activated SMASh tag [43]. However, if treatment with trimethoprim (TMP) (**7**) is applied, the DDs get stabilized, averting the degradation of the fused Cas9, thus keeping it active [47]. This is like the system of Davis et al. in the way that by binding a small molecule, Cas9 is activated [41]; however, in this case, it occurs through the inhibition of degradation of Cas9 so that it can provide its nuclease activity [43]. In such systems, only the dosage and timing of Cas9 can be controlled using the concentration and temporal exposure of the small molecules, e.g., ASV or TMP [47]. To add spatial controllability to the Cas9 activity, two PPGs were added to TMP [32]. The PPGs were introduced at the amine groups because, in the co-crystal structure of TMP and DHFR (PDB: 7R6G), it is visible that the amine groups of TMP are buried in the binding pocket of DHFR [48]. In an eGFP disruption assay with a sgRNA Plasmid targeting the *eGFP* gene and DHFR-fused Cas9, treatment with PPG-modified TMP **7 a** or **7 b** did not induce an observable loss of fluorescence. This leads to the conclusion that protected TMP indeed cannot bind to the DDs. Modified Cas9 is therefore left unstable and is quickly degraded by the proteasome. However, after an irradiation treatment of just a couple of minutes, loss of fluorescence was observable, meaning that the deprotection of the protected TMP into free TMP was possible and the binding ability to the DDs was restored. Following irradiation, inhibition of proteasomal degradation was gained, and Cas9 was kept active. Apart from controlling double-strand breaks and silencing genes with DHFR-modified Cas9, the expression of IL1RN could be influenced. For that, a dCas9 was modified with DHFR, and the transcriptional activator domains (VP64 and PP7) were attached. When treatment with protected TMP was applied, no leverage of expression was observable, meaning that protected TMP could not bind to the modified dCas9, leading to dCas9 being degraded. After treatment with light for 12 min, however, unprotected TMP was formed, which led to its binding to the modified dCas9. By binding, the degradation of dCas9 was prevented, resulting in an increase in IL1RN expression proportional to light exposure and compound concentration [32]. In total, the findings of Manna et al. provide a similar control of Cas9 activity to the system of Wu et al. by turning Cas9 active through treatment with a small molecule [43]. However, the system of Manna et al. not only provides temporal and dosage control but also spatial control through the inclusion of a light-activable pathway. Furthermore, they did not only gain control of double-strand breaks but also of dCas9-mediated gene activation [32]. The structure of TMP and its PPG-modified version are shown in Figure 10a, the co-crystal structure of TMP and DHFR in Figure 10b, and the working principle in Figure 10c [32,48].

**Figure 11:**
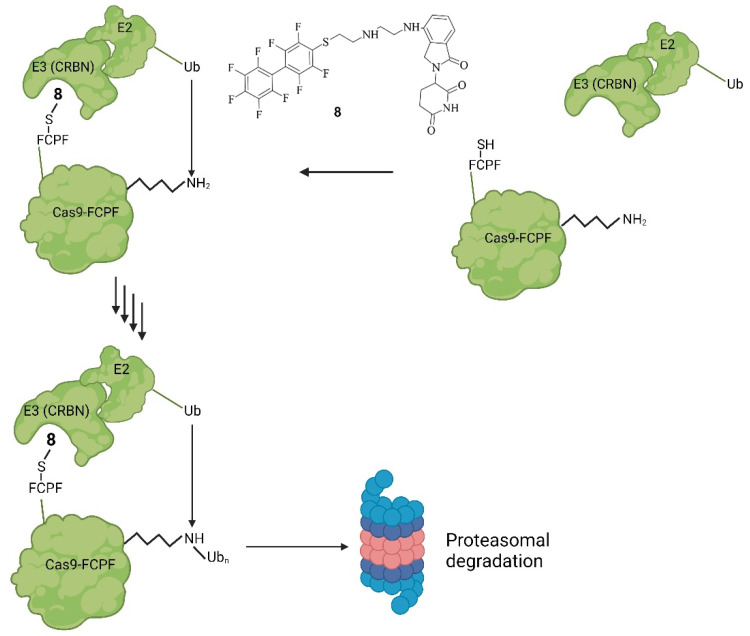
Mode of action of PROTAC-FCPF (**8**). Binding of **8** onto Cas9-FCPF leads to a complex with the E3-ligase CRBN. Attached to that is an E2-ligase. The ubiquitination of Cas9-FCPF is then catalysed, which ultimately leads to the degradation of Cas9-FCPF by the UPS, rendering Cas9-FCPF inactive [49,51].

In the systems described so far, rather substantial sequence modifications were needed to make Cas9 targetable by a small molecule. Recently, we modified Cas9 with an amino acid sequence consisting of phenylalanine, cysteine, proline, and phenylalanine (FCPF) [49]. This so-called π-clamp leads to a specific reactivity of the cysteine, which then reacts with perfluoro aromatic moieties [50]. With the FCPF modification of Cas9 (Cas9^FCPF^), small molecules could precisely recognize Cas9^FCPF^ [50]. This was used for labeling strategies, but most importantly for a proteolysis targeting chimera (PROTAC) [49]. PROTACs are hetero-bifunctional molecules with a ligand that binds an E3-ligase. The E3 ligand is connected via a linker to another ligand on the other side that can bind to the protein of interest (POI). By binding on both sides simultaneously, they can catalyze the transfer of ubiquitin (Ub) onto the POI. Ub is transferred from an E2 ligase, bound to the E3 ligase, which itself is bound to the POI. PROTAC serves as an enhancer of the binding between the E3 ligase and POI. Through the ubiquitination of the POI at a lysine residue or the N-terminal, it is marked for the 26S proteasome and is then degraded by the ubiquitin-proteasome system (UPS) [51]. We generated a perfluoro derivative conjugated with lenalidomide, a ligand of the E3-ligase Cereblon (CRBN), called PROTAC-FCPF (**8**). **8**-FCPF-Cas9 could be degraded in HeLa cells at a concentration of 10 µM after 6 h [49]. Via the T7E1 [52,53] assay, it was further proven that the biologic activity of Cas9^FCPF^ was comparable to unmodified Cas9. Degradation was further proven for dCas9^FCPF^, Cas12^FCPF^, and Cas13^FCPF^ [49]. In short, a similar system to that of Wu et al. was established. In both systems, Cas9’s stability is controlled by small-molecule-induced proteasomal degradation [43,49]. However, instead of introducing two domains as a SMASh-tag [43], only a peptide consisting of four amino acids was needed for Cas9^FCPF^ [49]. The mode of action of PROTAC-FCPF and its structure are shown in Figure 11 [49,51]. 

## 6. Small Molecules Regulate DSB Repair Mechanisms

**Figure 12:**
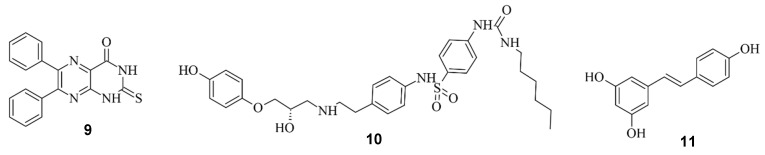
Scr7 (**9**), L755507 (**10**) and Resveratrol (**11**) from left to right.

Aside from regulating the activity of engineered Cas9, there is the possibility of modulating the CRISPR/Cas9 system by regulating the DNA repair system. Li et al. investigated three compounds regarding their ability to enhance HDR or down-regulate NHEJ towards a more precise editing [17]. In their study, Scr7 (**9**), L755507 (**10**), and Resveratrol (**11**) were found to inhibit DNA ligase IV and thereby reduce NHEJ-mediated repair [17], showing that the efficiency of knock-ins can be enhanced in the presence of chemical inhibitors targeting NHEJ-influencing factors [54]. The compounds are illustrated in Figure 12. 

**Figure 13:**
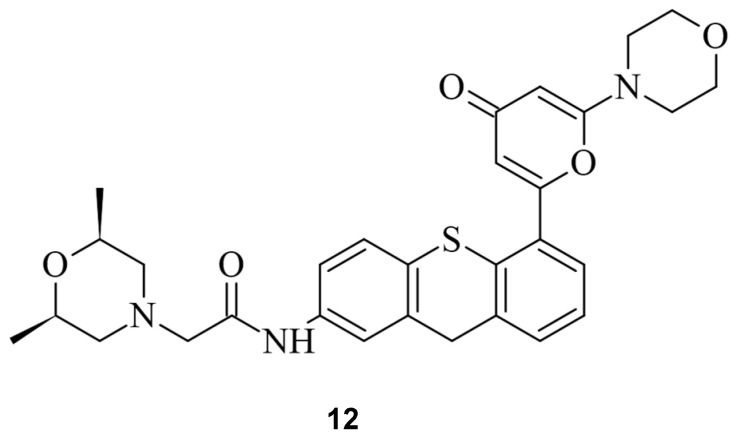
The ATM inhibitor KU60019 (**12**).

A similar approach was undertaken by Bermudez Cabrera et al. Multiple small molecules were investigated, with a focus on the Ataxia-telangiectasia mutated (ATM) inhibitor KU60019 (**12**), as illustrated in Figure 13 [55].

**Figure 14:**
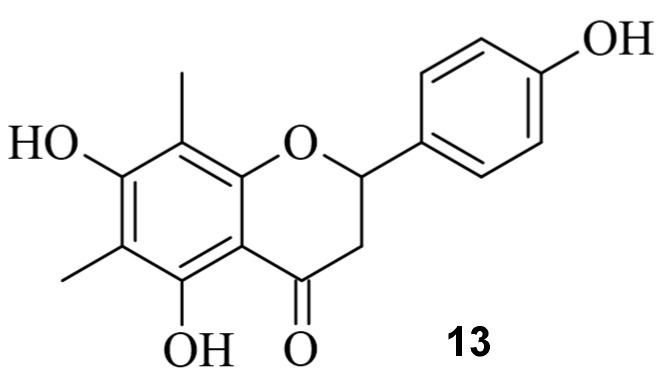
The small molecule farrerol (**13**).

ATM, a serine/threonine kinase, plays a determining role in the initiation of the DSB repair mechanism by recruiting necessary repair factors and determining whether HDR or NHEJ occurs [56]. During a study, a dose-dependent downshift from MH deletions (down to 0.74-fold) in the presence of various chemical compounds, including **11**, was observed. Also, an increase in editing efficiency was noticeable [55]. However, as the main repair mechanism of DSB, downregulating NHEJ is associated with increased potential for tumorigenesis [57,58,59]. To avoid side effects, Zhang et al. screened 722 small molecules and identified farrerol (**13**), which increased knock-in efficiency by up to 2.9-fold at 5 µM, while NHEJ was clearly not affected. Also, single-strand annealing (SSA), which is said to cause loss of genome integrity, could be downregulated by up to 3.3-fold at a concentration of 10 µM (Figure 14) [60,61]. 

## 7. Small Molecules Regulate sgRNAs

**Figure 15:**
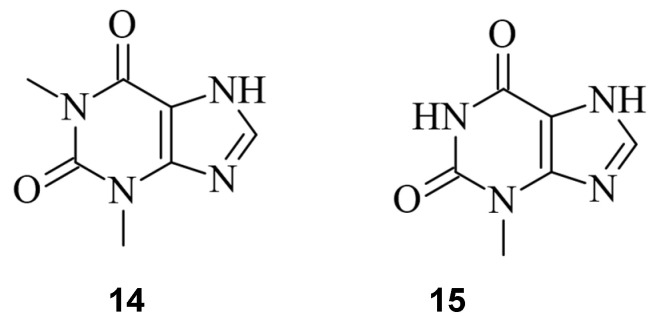
The RNA aptamer ligands theophylline (**14**) [64] and 3MX (**15**) [65].

Given the important role of sgRNAs in CRISPR/Cas9 [2], chemicals capable of controlling the activity of the sgRNA offer another possibility to regulate the process of genome editing [41,43]. Aptamers are short nucleic acids that can specifically bind ligands and are commonly employed to regulate nucleic acids, such as sgRNA [62]. Iwasaki et al. applied this concept to develop so-called aptamer-sgRNA (agRNA), in which the theophylline (**14**) or 3MX (**15**) aptamer was used and introduced into sgRNA at different positions, leading to an endonuclease-active Cas9 only upon treatment with **14** or **15**, respectively. An up to 10^4^-fold increase in transformants in comparison to wild-type sgRNA was observed (Figure 15) [63]. 

**Figure 16:**
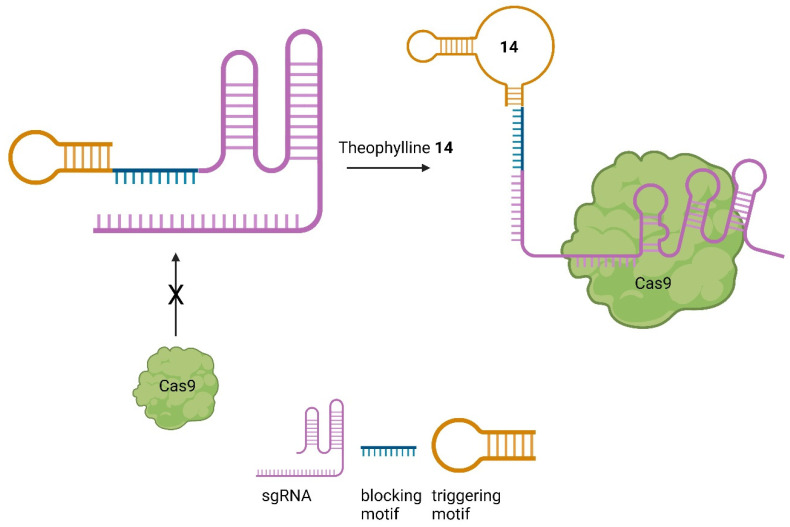
Mode of action of SMART-sgRNAs [66].

Based on the theophylline aptamer, Bingqian et al. designed small-molecule-activated allosteric aptamer-regulating (SMART)-sgRNAs [66]. Instead of inhibiting the nuclease activity of Cas9, as demonstrated by Iwasaki et al. [63], they used a blocking motif to inhibit the binding of Cas9 in the first place. Further, a triggering motif at the sgRNA 3′ end was attached, containing the theophylline aptamer. In the absence of theophylline, the blocking and triggering motifs form a loop to block Cas9 from binding. After treatment with theophylline, the structure is changed, which allows Cas9 to bind to the sgRNA, leading to the DSB (Figure 16) [66].

By employing this system, the cleavage of *EGFP* could be reduced in the absence of theophylline. Significantly so, in comparison to unmodified sgRNA. Moreover, the system was successfully applied to the firefly luciferase and TurboRFP genes by modifying the sgRNA while retaining the blocking and triggering motif. After treatment with theophylline, a SMART-sgRNA activation of up to 61% was observed. This system has been demonstrated to be effective both in vitro and in vivo, providing a versatile tool for activating sgRNA and thereby controlling Cas9 [66].

## 8. Downsides of Small Molecules and Ethical Viewing Points

Besides the examples of highly promising usages of small molecules to increase the specificity and efficiency of CRISPR/Cas9-based systems, small molecules are always accompanied by concerns and disadvantages. First, the toxicity and off-target effects of small molecules should always be in mind. The presented systems were all tested and quantified in cells, not in living organisms or even humans. In light of that, the off-target effects and selectivity in a larger biological system than cells for each chemical can be severely harmful [67]. In the presented case, especially if the main DSB repair mechanism of NHEJ is downregulated in an uncontrolled manner [57,58,59]. Downregulation of core NHEJ factors, for example, is known to occur in, e.g., colon cancer [68]. A very prominent example of an underestimated, or better said, not foreseen, side effect is the malformations caused by thalidomide back in the 1960s [69]. However, in recent years, new technologies to improve selectivity for specific cells have been invented. Based on nano particles (NP), e.g., cancer cells can be addressed more precisely [70]. With NP-based strategies to deliver CRISPR/Cas9 systems for genome editing present [71], it would be a highly attractive possibility to use NP-based delivery methods in singular NPs that can deliver the CRISPR/Cas system and the small molecule at once to certain types of cells. In that way, the precision of CRISPR/Cas9 technologies could be enhanced on two frontiers: delivery and controllability. Second, the applications of small molecules in vivo can always differ because of pharmacokinetics, solubility, etc [67]. Water solubility and therefore pharmacological activity are highly dependent on ionization which is in turn dependent on the pH value. This value can highly alternate between 1.5 and 8 in the human body, alternating solubility and therefore working efficiency of small molecules [72,73]. For example, 4-HT (Section 5) has a rather big lipophilic structure consisting of three aromatic rings. Such structures tend to display poor water solubility, leaving them with bad delivery and pharmacological-acting properties [74]. From another, highly important point of view, the CRISPR/Cas-technologies provide some downsides within their goal to modify genes or whole genetics as well. The question opens itself up about where to stop and where to start with genetic engineering. What could be a disease for one person could be a unique trait in the eyes of another person. Conditions like down syndrome even have beneficial aspects, like a higher resistance to certain types of tumours [75]. Therefore, the scientific and sooner or later, the general community should be aware of the power that emerges with new technologies.

## 9. Conclusions

The CRISPR/Cas9 system is a promising tool in gene editing with multiple thinkable applications. The therapeutic applications may be the most interesting [3]. To use the system in the best way possible in terms of, e.g., efficiency and non-toxicity, control over dosage, timing, and spatial action is unavoidable [6,32]. Small molecules offer a great possibility to regulate Cas9s activity because of their advantages of relatively low price, often oral bioavailability, simple chemical synthesis, broad modification possibilities, and so on [36]. Especially in comparison to the relatively expensive and otherwise complicated handling of Cas9-controlling therapeutic proteins [35]. In this review, a broad spectrum of small molecules that could influence Cas9-mediated gene editing is presented. A relatively large proportion of them interact with modified Cas9 variants and can either activate or deactivate Cas9 [32,41,43,49]. Further, control over the activity and interaction with modified sgRNA [63,66] was achieved. Also, regulation of the repair mechanisms following a DSB was shown to be possible [17,55,61]. But not only the activity of modified Cas9 could be altered by small molecules. Some molecules were proven to even downregulate Cas9^wt^ [38,47]. However, not only indels with Cas9 are controllable [14,15]. Gene activation using endonuclease lacking dCas9 with the use of small molecules was further achievable [32]. All in all, multiple ways of controlling the effects of Cas9s were displayed. All these methods provide promising tools for turning Cas9-based technologies safer and more efficient. Either by decreasing unwanted effects, e.g., off-target editing, or enhancing on-target efficiency through, e.g., more precise activation [6,31,47]. In the future, such systems can have big impacts in medicine and chemistry. Perhaps by even combining the ideas and principles of the individual techniques, e.g., using NPs to deliver CRISPR/Cas9 systems and NPs simultaneously to certain types of cells [70,71]. However, as discussed in the last section, the risks of small molecules [67] should always be considered, and their applications should be well thought through before being applied to living creatures, especially human. What can be the solution or cure for one disease can be the origin of another, perhaps way more drastic condition [67,69].
